# Exploration of Potential Roles of m6A Regulators in Colorectal Cancer Prognosis

**DOI:** 10.3389/fonc.2020.00768

**Published:** 2020-05-15

**Authors:** Liechen Ji, Shuo Chen, Liqiang Gu, Xipeng Zhang

**Affiliations:** Department of Colorectal Surgery, Tianjin Union Medical Center, Nankai University, Tianjin, China

**Keywords:** colorectal cancer, m6A, m6A regulators, prognosis, TCGA

## Abstract

Colorectal cancer (CRC) represents one of the most common malignancies with high morbidity worldwide. RNA methylation (m6A) has been considered to tremendously contribute to cancer initiation and progression since its first discovery. In this study, we comprehensively analyzed associations between mRNA expressions of m6A regulators and CRC tumor samples' epidemiologic information from the Cancer Genome Atlas (TCGA). Multivariate Cox proportional hazard model was applied to screening of m6A regulators whose mRNA expressions were significantly associated with CRC tumor samples' overall survival (OS) probability and those significant regulators were used for LASSO regression analysis to construct CRC prognosis prediction signature. As a result, two regulators i.e., YTHDC2 and ALKBH5 were picked out in multivariate analysis. CRC prognosis signature was constructed based on those two regulators through which CRC tumor samples with favorable and inferior prognosis could definitely be distinguished independent of potential confounding factors. This study should be helpful for identifying prognostic different CRC patients and guiding therapeutic method selection.

## Introduction

According to the global burden of disease study in 2018, colorectal cancer (CRC) is one of the most common cancer types worldwide, and represents the third leading cause of cancer-related deaths (1). Many risk factors have been associated with CRC, including genetic and environmental factors. Hereditary CRC accounts for ~5–10% of all CRC cases, while the tumorigenesis and development of CRC arise through the accumulation of a multistep carcinogenic process that affected by lifestyle and dietary factors (2). In the last decade, increasingly researches have focused on the multiple molecular pathways involved in CRC pathogenesis, especially genetic and epigenetic events (3, 4). It is generally believed that, epigenetic alterations in CRC occur earlier and happened more frequently than genetic alterations (5, 6). Genomic instability, mutational inactivation of tumor suppressor genes, and activation of oncogenes are all involved in its development (6). Nowadays, genome-wide association studies have recently linked CRC to several common genetic variants or single-nucleotide polymorphisms, meanwhile, miRNA profiling of CRCs has identified over 20 up and downregulated miRNAs (7).

Natural RNA molecules contain multiple chemically modified nucleosides (8). Aside of DNA methylation and histone modification, mRNA modification represents another layer of epigenetic regulation of gene expression (9). The extent of RNA modifications is highly sensitive to changes in cellular micro-environment or the switch between different physiological states, and these changes in patterns of RNA modification would in turn affect the regulation for cell function and adaptation (10, 11). A wide range of RNA base modifications, collectively called the epitranscriptome, have been implicated in translation control, RNA splicing defects and many cancer types (12). N6-methyl-adenosine (m6A) is the prevalent modification in eukaryotic mRNAs, whose reversible methylation may have a profound impact on gene expression regulation (13). Abnormal methylation of m6A would cause a range of diseases, including tumors (14, 15), neurological diseases (16, 17), and embryonic retardation (18). Recently, their roles in cancer biology and cancer stem cells have become prominent hotspot in the research field of on tumorigenesis and screening of potential biological targets. However, the cellular and functional dynamics of m6A RNA modifications in the regulation of CRC development remain largely unexplored.

In this study, the epidemiologic information of 522 CRC samples was obtained from the Cancer Genome Atlas (TCGA). We analyzed the association between the expression of m6A regulators and CRC initiation and progression, screened the key regulators whose expression was related with the overall survival (OS) of CRC patients, and constructed a CRC prognosis signature based on these regulators to evaluate the roles of m6A regulators in CRC development and prognosis.

## Materials and Methods

### Study Population

All subjects used in this study were from the Cancer Genome Atlas (TCGA). A total of 522 samples including 487 CRC tumor tissues and 35 adjacent normal tissues were included. Detailed epidemiological characteristics of those CRC patients are provided in Table 1.

**Table 1:** Clinicopathological characteristics of COAD patients from TCGA database.

**Characteristics**	**COAD patients (*****N*** **=** **487)**	
	**NO**.	**%**
**AGE**		
≤ 68(Median)	243	49.90
>68(Median)	244	50.10
**GENDER**		
Female	229	47.02
Male	258	52.98
**RACE**		
White	238	48.87
Black or African American	62	12.73
Asian	11	2.26
American Indian or Alaska	2	0.41
Unknown	174	35.73
**PATHOLOGIC STAGE**		
i	83	17.04
ii	196	40.25
iii	136	27.93
iv	72	14.78
**SURVIVAL TIME**		
Long (>5 years)	45	9.24
Short (<5 years)	442	90.76
**OS STATUS**		
Dead	111	22.79
Alive	376	77.21

### m6A Regulators

A total of 12 m6A regulators containing 5 writers (methyltransferase like 3, METTL3; methyltransferase like 14, METTL14; WT1-associated protein, WTAP; RNA binding motif protein 15, RBM15; and zinc finger CCCH domain-containing protein 13, ZC3H13), 5 readers (YTH domain-containing 1, YTHDC1; YTH domain-containing 2, YTHDC2; YTH N6-methyladenosine RNA binding protein 1, YTHDF1; YTH N6-methyladenosine RNA binding protein 2, YTHDF2; and heterogeneous nuclear ribonucleoprotein C, HNRNPC) and 2 erasers (fat mass- and obesity-associated protein, FTO; α-ketoglutarate-dependent dioxygenase alkB homolog 5, ALKBH5) were collected in this study. MRNA expressions of those regulators in CRC tumor and adjacent normal tissues from TCGA were also obtained for constructing CRC prognosis prediction model.

### Construction of Prognosis Prediction Model

Multivariate Cox regression analysis was performed for screening regulators whose mRNA expressions significantly correlate with CRC tumor samples' overall survival (OS) probability. LASSO Cox regression was applied to those significant regulators for development of potential CRC prognosis signatures.

### Survival Analysis

OS probability of every CRC tumor sample was estimated by Kaplan-Meier method. Log-rank test was used to determine the significance of OS probability between different categorical CRC groups. Multivariate Cox proportional hazard model was applied to adjust influences of confounding factors on CRC samples' prognosis.

### Nomogram Analysis

We used rms R package to perform nomogram analysis by including those factors that significantly associated with OS of CRC patients in multivariate analysis. Calibration plot was applied to estimate the discrimination between actual and nomogram predicted OS probability.

### Statistical Analysis

Kolmogorov-Smirnov (K-S) test was used for normality test of mRNA expressions across CRC samples. Wilcoxon ran test or *t*-test was applied to the comparison of mRNA expressions between CRC tumor and adjacent normal samples. Comparisons of mRNA expressions among more than two groups were performed by analysis of variance (ANOVA). All the statistical analysis was conducted in R 3.4.3. *P* < 0.05 was used as the significant threshold.

## Results

### Associations Between m6A Regulators and CRC Initiation and Progression

**Figure 1:**
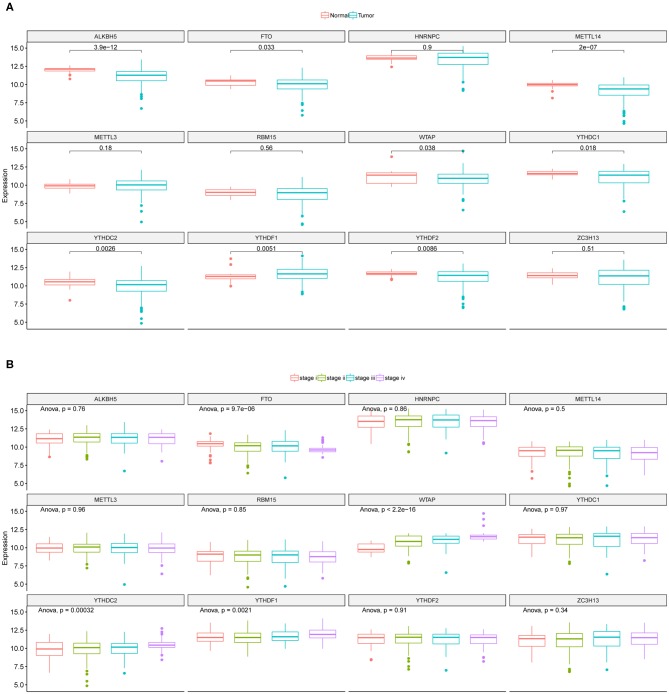
Expression landscape of m6A regulators across CRC samples. **(A)** Boxplots illustrating m6A regulators' expression values across CRC tumor and adjacent normal tissues along with *t*-test *p*-value provided above the boxplot. **(B)** Boxplots illustrating the expression levels of the 12 m6A regulators across CRC tumor samples with different stages. ANOVA *p*-values were provided above the boxplot.

The mRNA expressions of the 12 m6A regulators in CRC tumor and adjacent normal samples from TCGA were obtained and illustrated as boxplots (Figure 1A). Comparisons between adjacent normal and tumor samples identified seven down-regulated (METTL14, WTAP, YTHDC1, YTHDC2, ALKBH5, FTO, and YTHDF2) and one up-regulated (YTHDF1) regulators. CRC tumor samples were further divided into different stages, i.e., stage I-IV, and the 12 regulators' mRNA expressions were provided as boxplots (Figure 1B). ANOVA for the comparison of every regulator's mRNA expression among different stages illustrated that WTAP and FTO exhibited significantly sustained elevated and decreased mRNA levels with the progression of CRC, respectively. Table S1 provided the mRNA levels of those 12 m6A regulators in the CRC tumor samples used in this study. Those results indicated the potential associations between some of the m6A regulators and CRC initiation and progression. So, they should be feasible for the following analysis for screening of CRC prognostic signature.

### Association Between m6A Regulators and CRC Prognosis

**Figure 2:**
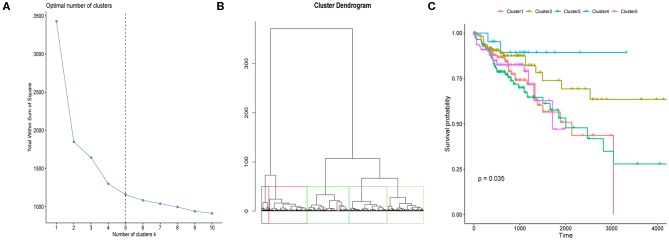
Clustering of CRC tumor samples and prognosis analysis. **(A)** Line chart used for the determination of the optimal sample cluster number for hierarchical clustering analysis of CRC tumor samples. Horizontal and vertical axis represents cluster number (k) and total within sum of square, respectively. **(B)** Hierarchical clustering of CRC tumor samples based on the 12 m6A regulators' expression values. **(C)** Kaplan-Meier curves of CRC tumor samples stratified by their cluster information with log-rank test *p*-value provided.

We here proposed to explore if CRC tumor samples could be distinguished with respect to their prognosis based on the eight differentially expressed regulators' mRNA expressions. We first determined the optimal sample clustering number as five through consensus clustering analysis as shown in Figure 2A. Euclidean distance among the CRC tumor samples were calculated based on the eight regulators' mRNA expressions which was then applied to hierarchical clustering method. Figure 2B illustrated the tumor sample clustering result. Samples allocated to the five clusters exhibited significantly different OS probability as shown in Figure 2C which suggested potential association between m6A regulators and CRC prognosis.

### m6A Regulator-Based CRC Prognosis Signature

**Figure 3:**
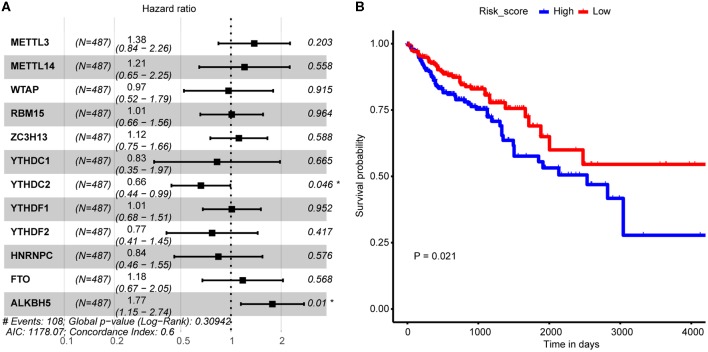
Association between m6A regulator and CRC tumor samples' prognosis. **(A)** Forest plot shows the result of multivariate Cox-regression analysis for association between m6A regulator's expression and Kaplan-Meier estimated CRC tumor samples' overall survival probability. Values within brackets represent 95% confidence interval of hazard ratio. **(B)** Kaplan-Meier curves of CRC tumor samples stratified by the median risk score with log-rank test *p*-value provided.

The above analysis implied potential application of combination of the 12 m6A regulators in CRC OS probability prediction. In consideration of both cost-efficiency and accuracy, we proposed to further screen m6A regulators that significantly correlated with CRC OS probability through multivariate Cox proportional hazard model. As a result, YTHDC2 and ALKBH5 were picked out (Figure 3A) which were then applied to LASSO regression analysis to construct CRC prognosis prediction signature. Risk score of every CRC tumor sample was calculated, and samples were then stratified by the median risk score. Log-rank test uncovered distinctly different OS probability between the two sample groups (Figure 3B), which should suggest the reliability of our signature in CRC prognosis prediction.

### Associations Between Risk Score and CRC Epidemiology Statistics

**Figure 4:**
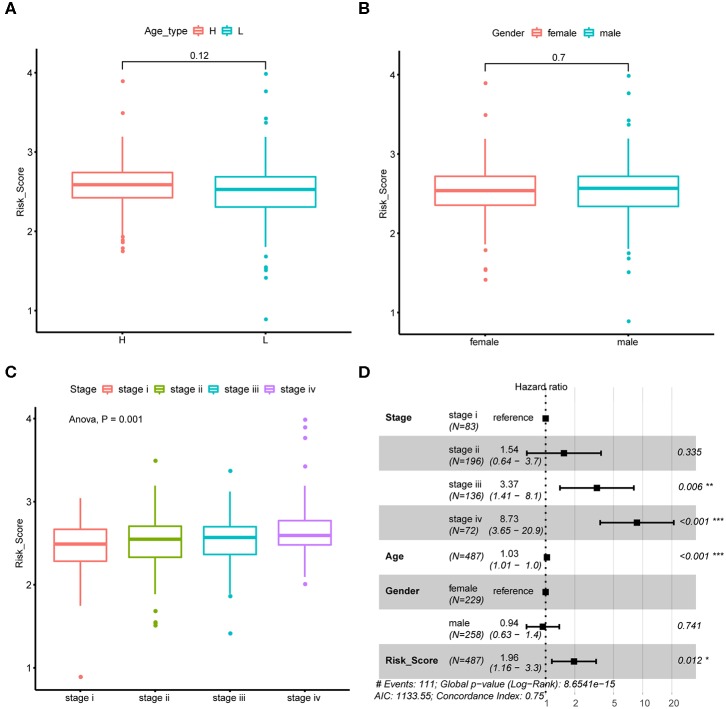
Association between risk score and CRC tumor samples' epidemiologic information. **(A)** Boxplots show the distribution of CRC tumor samples' risk score stratified by the sample's median age. **(B)** Boxplots show the distribution of CRC tumor samples' risk score stratified by sample's gender. **(C)** Boxplots show the distribution of CRC tumor samples' risk score stratified by tumor stage. **(D)** Forest plot shows the result of multivariate Cox-regression analysis for association between risk score and Kaplan-Meier estimated CRC tumor samples' overall survival probability after adjusted age, gender and stage.

We next explore if CRC tumor samples' risk scores were correlated with their common epidemiology statistics which mainly involved age, gender, and stage. As a result, risk scores of samples were not significantly different between elder and younger patients when stratified by the median age (Figure 4A) as well as between male and female patients (Figure 4B). While, risk score kept rising through stage I to stage IV as shown in Figure 4C which was consistent with its unfavorable prognosis role. Besides, multivariate Cox regression analysis suggested that the m6A regulator-based signature could reliably predict CRC patients' prognosis independent of age, gender, and stage (Figure 4D).

### Construction and Validation of Nomogram

**Figure 5:**
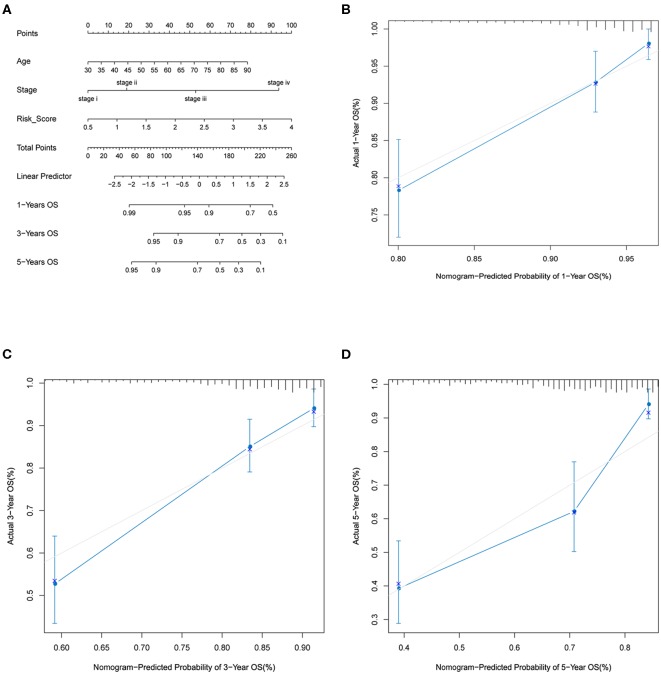
Nomogram analysis. **(A)** Nomogram composed of age, stage, and risk score for the prediction of 1-, 3-, and 5-years OS probability. Calibration plot for the evaluation of the nomogram in predicting 1-year **(B)**, 3-years **(C)**, and 5-years **(D)** OS probability.

By including significant factors in multivariate Cox-regression analysis, i.e., age, stage, and risk score, we constructed a nomogram to predict 1-, 3-, and 5-years OS probability as shown in Figure 5A. Calibration plot indicated that nomogram-predicted OS probability deviated very little from actual OS probability, particular for 1- and 3-years OS probability (Figures 5B–D). Those should suggest the potential of the risk score as a reinforcement for epidemiological features to improve the estimation of CRC prognosis.

## Discussion

Similar to DNA methylation and histone modification, m6A RNA modifications can be added by writer enzymes and removed by eraser enzymes. The differential expressions of specific RNA m6A methylation regulators are linked to mis-regulated RNAs in tumors, however, the same m6A methylation regulators may have distinct functions in different tumors (19). In this study, we proposed to explore the feasibility of the common m6A regulators in CRC prognosis estimation. Differential expression analysis found that 8 m6A regulators, including METTL14, WTAP, YTHDC1, YTHDC2, ALKBH5, FTO, YTHDF1, and YTHDF2, were significantly differently expressed between adjacent normal and tumor samples. This should preliminarily suggest that those m6A regulators have the potential of influencing CRC initiation and could be used for the subsequent analysis. In addition, among those differential expression regulators, the expressions of WTAP and FTO exhibited significantly sustained elevated and decreased with the progression of CRC, respectively, suggesting enhanced m6A RNA methylation with the increasing tumor grade. METTL3 and METTL14 were both writer m6A regulatory enzymes, and WTAP can improve methylation efficiency in nuclear speckles by translocating the METTL3-METTL14 complex to mRNA targets (20). FTO is the first discovered m6A eraser that can influence the transcription of adjacent genes (21, 22). The up-regulation of WTAP was able to promote the proliferation and survival of tumor cells, while FTO plays an oncogenic role as an m6A demethylase in acute myeloid leukemia (22, 23). Furthermore, the expression trend of WTAP and FTO that same as our result was positively associated with the malignant progression in gliomas (24).

The abnormal methylation of m6A mRNA has shown prognostic value in multiple tumors, such as cervical cancer (25), acute myeloid leukemia (26), pancreatic cancer (27), and hepatocellular carcinoma (28). Kandimalla's group has reported a risk-score derived from a seven gene mRNA expression classifier consisting of METTL3, METTL14, WTAP, YTHDF1, YTHDF2, FTO, and ALKBH5 that associated with poor disease-free survival of CRC patients (29). In this study, YTHDC2 and ALKBH5 were picked out by multivariate Cox proportional hazard model, and applied for the construction of CRC prognosis prediction signature. The result of multivariate Cox regression analysis suggested that the signature based on YTHDC2 and ALKBH5 was able to predict CRC patients prognosis reliably independent of age, gender and stage.

YTHDC2 is a nuclear localized protein that is widely expressed in human cancer cell lines (30, 31). YTHDC2 can promote cancer metastasis via enhancing the translating efficiency of hypoxia-inducible factor (HIF)-1α in colon tumor cells, and may become a diagnostic marker and target gene for treating colon cancer patients (32). Fanale et al. suggested YTHDC2 as a potential candidate for pancreatic cancer susceptibility and a useful marker for early detection (32). However, the regulatory mechanism of YTHDC2 on tumor behavior is still poorly understood. ALKBH5 belongs to the non-heme Fe(ii)- and α-ketoglutarate (KG)-dependent dioxygenase AlkB family of proteins demethylated NANOG mRNA, and stimulated the expressions of HIF-1α and HIF-2α of breast cancer cells when exposure to hypoxia (33, 34). It was reported that, the growth of CRC is associated with the physiological state of hypoxia, and HIF-1α is a key factor for CRC metastasis (35). What's more, aberrant expressions of YTHDC2 and ALKBH5 in the context of CRC initiation and progression have been previously reported, and consistent results as the current study were obtained (32, 36). Therefore, we speculate that, the RNA methylation regulated by YTHDC2 and ALKBH5 might regulate tumor proliferation and metastasis through HIF-mediated hypoxic microenvironment response and then affect the prognosis of patients, which would be confirmed in our future research.

## Conclusion

In this study, we comprehensively analyzed associations between mRNA expressions of m6A regulators with the development and prognosis of CRC. Among the m6A regulators, the abnormal expressions of WTAP and FTO were found to be significantly related to the progression of CRC, and YTHDC2 and ALKBH5 were identified as key regulators that could predict the prognosis of patients with CRC independently. This study highlighted the important role of RNA modification in the development of CRC, and provided potential guiding biomarkers for the therapeutic method selection.

## Data Availability Statement

Publicly available datasets were analyzed in this study. This data can be found at: The Cancer Genome Atlas (TCGA), Datasets link: https://portal.gdc.cancer.gov/repository.

## Author Contributions

LJ and SC put forward the ideas of this article, wrote this article, and analyzed the data. LG helped with acquisition of data and analysis and interpretation of data. XZ analyzed the data and helped with revising the manuscript. All authors read and approved the final manuscript.

## Conflict of Interest

The authors declare that the research was conducted in the absence of any commercial or financial relationships that could be construed as a potential conflict of interest.

## References

[1] Global Burden of Disease Cancer C Fitzmaurice C Akinyemiju TF Al Lami FH Alam T Alizadeh-Navaei R. Global, regional, and national cancer incidence, mortality, years of life lost, years lived with disability, and disability-adjusted life-years for 29 cancer groups, 1990 to 2016: a systematic analysis for the global burden of disease study. JAMA Oncol. (2018) 4:1553–68. 10.1001/jamaoncol.2018.2706 29860482 PMC6248091

[2] Tanaka T. Colorectal carcinogenesis: review of human and experimental animal studies. J Carcinog. (2009) 8:5. 10.4103/1477-3163.49014 19332896 PMC2678864

[3] Lao VV Grady WM. Epigenetics and colorectal cancer. Nat Rev Gastroenterol Hepatol. (2011) 8:686–700. 10.1038/nrgastro.2011.173 22009203 PMC3391545

[4] Zoratto F Rossi L Verrico M Papa A Basso E Zullo A. Focus on genetic and epigenetic events of colorectal cancer pathogenesis: implications for molecular diagnosis. Tumour Biol. (2014) 35:6195–206. 10.1007/s13277-014-1845-9 25051912

[5] Baylin SB Ohm JE. Epigenetic gene silencing in cancer - a mechanism for early oncogenic pathway addiction? Nat Rev Cancer. (2006) 6:107–16. 10.1038/nrc1799 16491070

[6] Okugawa Y Grady WM Goel A. Epigenetic alterations in colorectal cancer: emerging biomarkers. Gastroenterology. (2015) 149:1204–25 e1212. 10.1053/j.gastro.2015.07.011 26216839 PMC4589488

[7] Goel A Boland CR. Recent insights into the pathogenesis of colorectal cancer. Curr Opin Gastroenterol. (2010) 26:47–52. 10.1097/MOG.0b013e328332b850 19786869 PMC2846600

[8] Cantara WA Crain PF Rozenski J Mccloskey JA Harris KA Zhang X. The RNA Modification Database, RNAMDB: 2011 update. Nucleic Acids Res. (2011) 39:D195–201.a 10.1093/nar/gkq1028 21071406 PMC3013656

[9] Liu N Pan T. RNA epigenetics. Transl Res. (2015) 165:28–35. 10.1016/j.trsl.2014.04.003 24768686 PMC4190089

[10] He C. Grand challenge commentary: RNA epigenetics? Nat Chem Biol. (2010) 6:863–5. 10.1038/nchembio.482 21079590

[11] Yi C Pan T. Cellular dynamics of RNA modification. Acc Chem Res. (2011) 44:1380–8. 10.1021/ar200057m 21615108 PMC3179539

[12] Li S Mason CE. The pivotal regulatory landscape of RNA modifications. Annu Rev Genomics Hum Genet. (2014) 15:127–50. 10.1146/annurev-genom-090413-025405 24898039

[13] Niu Y Zhao X Wu YS Li MM Wang XJ Yang YG. N6-methyl-adenosine. (m6A) in RNA: an old modification with a novel epigenetic function. Genomics Proteomics Bioinform. (2013) 11:8–17. 10.1016/j.gpb.2012.12.002 23453015 PMC4357660

[14] Wang S Sun C Li J Zhang E Ma Z Xu W Roles of RNA methylation by means of N(6)-methyladenosine (m(6)A) in human cancers. Cancer Lett. (2017) 408:112–20. 10.1016/j.canlet.2017.08.030 28867248

[15] Sun T Wu R Ming L. The role of m6A RNA methylation in cancer. Biomed Pharmacother. (2019) 112:108613. 10.1016/j.biopha.2019.108613 30784918

[16] Yoon KJ Ringeling FR Vissers C Jacob F Pokrass M Jimenez-Cyrus D Temporal control of mammalian cortical neurogenesis by m(6)A methylation. Cell. (2017) 171:877–89 e817. 10.1016/j.cell.2017.09.003 28965759 PMC5679435

[17] Chen X Yu C Guo M Zheng X Ali S Huang H. Down-Regulation of m6A mRNA Methylation Is Involved in Dopaminergic Neuronal Death. ACS Chem Neurosci. (2019) 10:2355–63. 10.1021/acschemneuro.8b00657 30835997

[18] Geula S Moshitch-Moshkovitz S Dominissini D Mansour AA Kol N Salmon-Divon M. Stem cells. m6A mRNA methylation facilitates resolution of naive pluripotency toward differentiation. Science. (2015) 347:1002–6. 10.1126/science.1261417 25569111

[19] Deng X Su R Feng X Wei M Chen J Role of N(6)-methyladenosine modification in cancer. Curr Opin Genet Dev. (2018) 48:1–7. 10.1016/j.gde.2017.10.005 29040886 PMC5869081

[20] Scholler E Weichmann F Treiber T Ringle S Treiber N Flatley A Interactions, localization, and phosphorylation of the m(6)A generating METTL3-METTL14-WTAP complex. RNA. (2018) 24:499–512. 10.1261/rna.064063.117 29348140 PMC5855951

[21] Loos RJ Bouchard C. FTO: the first gene contributing to common forms of human obesity. Obes Rev. (2008) 9:246–50. 10.1111/j.1467-789X.2008.00481.x 18373508

[22] Li Z Weng H Su R Weng X Zuo Z Li C FTO plays an oncogenic role in acute myeloid leukemia as a N(6)-methyladenosine RNA demethylase. Cancer Cell. (2017) 31:127–41. 10.1016/j.ccell.2016.11.017 28017614 PMC5234852

[23] Bansal H Yihua Q Iyer SP Ganapathy S Proia DA Penalva LO. WTAP is a novel oncogenic protein in acute myeloid leukemia. Leukemia. (2014) 28:1171–4. 10.1038/leu.2014.16 24413322 PMC4369791

[24] Chai RC Wu F Wang QX Zhang S Zhang KN Liu YQ m(6)A RNA methylation regulators contribute to malignant progression and have clinical prognostic impact in gliomas. Aging. (2019) 11:1204–25. 10.18632/aging.101829 30810537 PMC6402513

[25] Wang X Li Z Kong B Song C Cong J Hou J Reduced m(6)A mRNA methylation is correlated with the progression of human cervical cancer. Oncotarget. (2017) 8:98918–30. 10.18632/oncotarget.22041 29228737 PMC5716777

[26] Kwok CT Marshall AD Rasko JE Wong JJ Genetic alterations of m(6)A regulators predict poorer survival in acute myeloid leukemia. J Hematol Oncol. (2017) 10:39 10.1186/s13045-017-0410-6 28153030 PMC5290707

[27] Cho SH Ha M Cho YH Ryu JH Yang K Lee KH. ALKBH5 gene is a novel biomarker that predicts the prognosis of pancreatic cancer: a retrospective multicohort study. Ann Hepatobiliary Pancreat Surg. (2018) 22:305–9. 10.14701/ahbps.2018.22.4.305 30588520 PMC6295372

[28] Zhao X Chen Y Mao Q Jiang X Jiang W Chen J. Overexpression of YTHDF1 is associated with poor prognosis in patients with hepatocellular carcinoma. Cancer Biomark. (2018) 21:859–68. 10.3233/CBM-170791 29439311 PMC13078334

[29] Kandimalla R Gao F Ruan C Hsieh M Wang X Goel A Dysregulation of m6A RNA methylation regulators in colorectal cancer: clinical implication as prognostic biomarkers and potential therapeutic targets. Cancer Res. (2018) 78:3321 10.1158/1538-7445.AM2018-332129669760

[30] Fu Y Dominissini D Rechavi G He C Gene expression regulation mediated through reversible m(6)A RNA methylation. Nat Rev Genet. (2014) 15:293–306. 10.1038/nrg3724 24662220

[31] Jain D Puno MR Meydan C Lailler N Mason CE Lima CD. ketu mutant mice uncover an essential meiotic function for the ancient RNA helicase YTHDC2. Elife. (2018) 7:e30919. 10.7554/eLife.30919 29360036 PMC5832417

[32] Tanabe A Tanikawa K Tsunetomi M Takai K Ikeda H Konno J. RNA helicase YTHDC2 promotes cancer metastasis via the enhancement of the efficiency by which HIF-1alpha mRNA is translated. Cancer Lett. (2016) 376:34–42. 10.1016/j.canlet.2016.02.022 26996300

[33] Zheng G Dahl JA Niu Y Fedorcsak P Huang CM Li CJ. ALKBH5 is a mammalian RNA demethylase that impacts RNA metabolism and mouse fertility. Mol Cell. (2013) 49:18–29. 10.1016/j.molcel.2012.10.015 23177736 PMC3646334

[34] Zhang C Samanta D Lu H Bullen JW Zhang H Chen I Hypoxia induces the breast cancer stem cell phenotype by HIF-dependent and ALKBH5-mediated m(6)A-demethylation of NANOG mRNA. Proc Natl Acad Sci USA. (2016) 113:E2047–56. 10.1073/pnas.1602883113 27001847 PMC4833258

[35] Nagaraju GP Bramhachari PV Raghu G El-Rayes BF. Hypoxia inducible factor-1alpha: its role in colorectal carcinogenesis and metastasis. Cancer Lett. (2015) 366:11–8. 10.1016/j.canlet.2015.06.005 26116902

[36] Liu X Liu L Dong Z Li J Yu Y Chen X Expression patterns and prognostic value of m(6)A-related genes in colorectal cancer. Am J Transl Res. (2019) 11:3972–91. 31396313 PMC6684930

